# Chronic kidney disease in Nicaragua: a qualitative analysis of semi-structured interviews with physicians and pharmacists

**DOI:** 10.1186/1471-2458-13-350

**Published:** 2013-04-16

**Authors:** Oriana Ramirez-Rubio, Daniel R Brooks, Juan Jose Amador, James S Kaufman, Daniel E Weiner, Madeleine Kangsen Scammell

**Affiliations:** 1 Department of Preventive Medicine and Public Health, Universidad Autonoma de Madrid, C/Arzobispo Morcillo 4, Madrid, 28029, Spain; 2 Department of Epidemiology, Boston University School of Public Health, 715 Albany St. T4E, Boston, MA, 02118, USA; 3 Research Service and Department of Medicine, VA New York Harbor Healthcare System, Research Service (151), 423 East 23rd St, New York, NY, 10010, USA; 4 Division of Nephrology, Department of Medicine, Tufts Medical Center and Tufts University School of Medicine, 800 Washington Street,, Boston, MA, 02111, USA; 5 Department of Environmental Health, Boston University School of Public Health, 715 Albany St. T4W, Boston, MA, 02118, USA

## Abstract

**Background:**

Northwestern Nicaragua has a high prevalence of chronic kidney disease (CKD) of unknown cause among young adult men. In addition, frequent occurrence of urinary tract infections (UTI) among men and a dysuria syndrome described by sugarcane workers as “chistata” are both reported. This study examines health professionals´ perceptions regarding etiology of these conditions and their treatment approaches, including use of potentially nephrotoxic medications.

**Methods:**

Nineteen in-person semi-structured interviews were conducted in November 2010 among ten physicians and nine pharmacists practicing in the region.

**Results:**

Health professionals perceived CKD as a serious and increasing problem in the region, primarily affecting young men working as manual laborers. All interviewees regarded occupational and environmental exposure to sun and heat, and dehydration as critical factors associated with the occurrence of CKD. These factors were also considered to play a role in the occurrence of chistata in the region. Health professionals indicated that reluctance among workers to hydrate might be influenced by perceptions of water contamination. Symptoms often were treated with non-steroidal anti-inflammatory drugs (NSAIDs), diuretics and antibiotics. Physicians acknowledged that the diagnosis of UTI usually was not based on microbial culture and opined that the use of potentially nephrotoxic medications may be contributing to CKD.

**Conclusions:**

Interviews provided evidence suggesting that medications such as diuretics, antibiotics and NSAIDs are widely used and sold over the counter for symptoms that may be related to dehydration and volume depletion. These factors, alone or in combination, may be possible contributors to kidney damage. Acute kidney damage coupled with volume depletion and exposures including medications and infectious agents should be further evaluated as causal factors for CKD in this region.

## Background

Chronic kidney disease (CKD) is a serious and increasing global health problem. Treatment for its most severe form, end stage renal disease, with dialysis or transplant is currently not available in many parts of the world [1,2]. Major known risk factors for CKD include diabetes and hypertension [3,4]. However, in lower income countries CKD may be associated with chronic glomerulonephritis and interstitial nephritis, which are generally ascribed to infectious and parasitic agents [5]. In Central America, case reports and government statistics document high mortality due to CKD, particularly among younger men and in certain regions of the Pacific coast [6-8]. Community prevalence studies in Nicaragua and El Salvador are consistent with these mortality data and have attempted to assess associations with pharmaceutical, behavioral, environmental and occupational exposures [9-15]. However, the causes of the high prevalence of CKD remain unknown.

Our team has been working in northwestern Nicaragua since 2009 as part of a mediation process (termed the “Dialogue Table”) including the management of Nicaragua Sugar Estates Limited (NSEL), a major sugar producer in northwestern Nicaragua that operates the Ingenio San Antonio located in the town of Chichigalpa, and the Association of Chichigalpans for Life (ASOCHIVIDA), a group of approximately 2000 former NSEL workers and community members who are affected by CKD. The Dialogue Table was established in 2008 after ASOCHIVIDA filed a complaint with the Compliance Advisor Ombudsman (CAO), the independent office that handles complaints from communities against the World Bank Group’s private sector arm which had provided funding to NSEL. CAO formed the Dialogue Table in response to the complaint, and the participants determined that they wanted an outside scientific group to make an independent assessment of the epidemic. Our team led by the Boston University School of Public Health was selected by Dialogue Table participants.

Our research occurred in stages, beginning with a “scoping study” summarizing the available information on CKD in the region, identifying data gaps, and recommending research activities to address those gaps [16]. During the process of conducting the scoping study we learned from members of the Dialogue Table and from informal discussions with area physicians of the frequent occurrence of a set of symptoms referred to locally as “chistata,” characterized by painful urination and often accompanied by “kidney” and/or back pain, and the common diagnosis of urinary tract infections (UTIs) among young men. Our final list of potential causes was extensive [16]. We identified several feasible activities that would provide more information to evaluate these hypotheses [16,17].

Among the subsequent research activities, we conducted qualitative interviews in Chinandega and Leon (regions with the highest CKD mortality rates in Nicaragua) both with physicians who are likely to diagnose or treat chistata, UTI, and/or CKD and with retail pharmacists who are likely to fill prescriptions or sell medications to treat these conditions. Medication use was on our list of hypotheses because members of the Dialogue Table reported that use of analgesics, and potentially nephrotoxic antibiotics that would require prescriptions in many higher income countries, were common in Nicaragua and could be obtained without a prescription. Medications are a common cause of *acute* kidney injury and may be associated with CKD [18]. For example, non-steroidal anti-inflammatory drugs (NSAIDs) are a frequent cause or contributor to acute kidney failure in the setting of severe volume depletion or other nephrotoxins. Aminoglycosides (a class of broad spectrum antibiotics) also are common causes of acute kidney failure, with risk factors including preexisting kidney disease, concomitant nephrotoxic medication use, and dehydration/volume depletion.

### Study aims

While there had been a great deal of media and activism focused on CKD and potential causes in Nicaragua, we did not know the general knowledge, opinion or practice of physicians and pharmacists in the region. Our aims in this study were to: (1) increase our understanding of health professionals´ perceptions regarding CKD in the region (characteristics of the affected population, causal hypotheses, symptoms, diagnostic tools, treatment and prognosis); (2) determine whether further study of the relationship among hydration practices, diagnosis of UTI/chistata and use of medications is warranted; and (3) explore potential opportunities for public health interventions related to the CKD epidemic aimed at physicians and pharmacists. This was the first effort known of by our team to engage physicians and pharmacists in Nicaragua in a formal assessment of the CKD epidemic.

## Methods

Recruitment and interviews were conducted in November 2010. In order to select interviewees, we acquired a list of public or government health care institutions from the Ministry of Health and a registry of pharmacies in Chinandega and León. We also acquired a list of private health institutions or clinics where CKD patients may seek care from the Social Security System. Each of the two regions (Chinandega and Leon) contained one main referral hospital, 13 and 16 public health centers, 6 and 4 social security or private clinics, and 145 and 212 pharmacies, respectively. We selected physicians from the health care institutions in each region so as to achieve a diverse sample with regard to sex, location, type of institution, and specialty of physician, and then selected pharmacies based on proximity to the sampled health care facilities. We estimated that 10 physicians and 10 pharmacists would be a feasible number to interview given time and budget constraints, would be sufficient to reach data saturation (i.e., redundancy in results to the point where interviews are not providing new information), and would enable us to identify trends and themes in the data.

We prepared an interview guide that consisted of 34 and 24 open-ended questions asked of physicians and pharmacists, respectively (available upon request). Open-ended questions asked in qualitative studies are typically designed to learn the opinion, beliefs, experience and reported behavior of the interviewee without prescribing response options or leading interviewees to a particular answer. We developed the interview guide with our hypotheses in mind, to learn from interviewees their opinion about the strength of various hypotheses based on their experience, and also to learn from physicians and pharmacists about their own hypotheses, if any, regarding the causes of CKD in the region. We began with broad questions such as, “What can you tell me about CKD in Nicaragua?” following up with more specific questions such as: “Do you think CKD is a problem in Nicaragua?”, “Has the number of cases you see each year changed in the time you have been in practice?” and “How would you describe the affected population?” For each major topic, we started with a broad question, “What do you think are the causes of CKD in Nicaragua?” and then followed up with specific questions that might indicate their opinion of hypothesized causes. Questions asked of both groups addressed the following topics: 1) prevalence and causes of CKD in Nicaragua; 2) population most affected; 3) beliefs regarding dysuria-related symptoms, prevalence of UTI and sexually transmitted infections; and 4) knowledge regarding indications and risks associated with use of potentially nephrotoxic medications. Additionally we asked physicians about standards and criteria for diagnosis and treatment of CKD and the availability and cost of diagnostic tests and medications. Interviews included a series of follow-up questions or probes to elicit additional information on these topics if not volunteered. A draft of the physician interview guide was tested with a physician in Nicaragua associated with our study team to determine if the length, organization and Spanish translation were appropriate.

Interviews were conducted individually and in-person by a native Spanish speaker (ORR). Informed consent was obtained at the time of the interview. Ethics approval was obtained both from the Institutional Review Board of Boston University Medical Campus and the Nicaraguan Ministry of Health. All interviews were electronically recorded, transcribed and translated into English by a professional transcription agency and cross-checked for accuracy by the interviewer. All identifiers (i.e., names of individuals, locations and names of institutions) were removed from English and Spanish versions of the transcripts prior to analysis. To protect anonymity, all research participants are referred to by a masculine pronoun regardless of sex.

Interviews were analyzed using standard social science methods for analyzing qualitative data [19]. An initial list of 33 codes (words and phrases used to tag portions of the transcript) was created based on the interview questions, which were based on existing hypotheses [20]. Coding the transcripts enables analysts to retrieve codes and associated data and to assign values of frequency, presence/absence and relationship with other codes [21]. For example, the code “CKDOCC” allowed us to tag and retrieve all text, including positive and negative examples, in which the occupation of CKD patients is referred to either in response to the question about the populations that are most affected, or in a different part of the interview. Text associated with each code can then subsequently be analyzed, and additional codes developed (e.g., OCCAG, to tag more specific mentions of agricultural occupation) [20]. Two analysts (MKS, ORR) separately coded the text and then together reviewed the codes. After agreement was reached on coded text, each analyst prepared summaries of the findings and met again to test agreement. The last step was to organize codes under thematic or conceptual headings and to assign meaning to the codes [20]. Specifically, we sought to understand how potential causes of the CKD epidemic identified in the interviews compared with our pre-existing hypotheses, and how perception of these causes might potentially inform future research activities.

## Results

**Figure 1:**
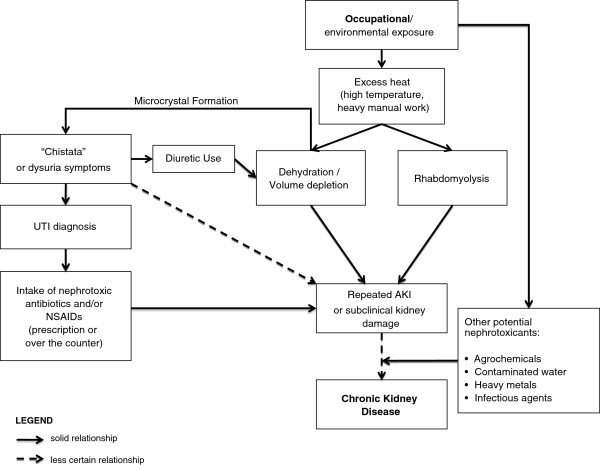
Conceptual model of excess heat hypothesis.

All physicians and pharmacists approached by our team agreed to be interviewed, demonstrating a high level of awareness and concern regarding the disease, societal effects and implications for their practice. We interviewed ten physicians and nine pharmacists who were diverse with regard to geographic areas and locations (Table 1). Six of the nine pharmacists reported a degree in Pharmacy and/or Chemistry, with three reporting no formal training. The results of our analysis are organized by headings that reflect key findings of physician and pharmacist opinions, experiences and practices. (See Additional file 1 for further quotes). Figure 1 demonstrates the relationships, with different degrees of certainty, identified by interviewees regarding several key causal hypotheses, beginning with the relationship between occupational exposure to heat and manual labor, followed by the occurrence of Chistata and diagnosis of UTI, use of medications, and eventual CKD, while including other potentially nephrotoxic exposures in what is largely considered to be a multi-factorial disease.

**Table 1:** Characteristics of interviewees

	**Physicians**	**Pharmacists**
	**n=10**	**n=9**
**Sex**		
Women	6 (60)	8 (89)
Men	4 (40)	1 (11)
**Region**		
Leon	5 (50)	5 (56)
Chinandega	5 (50)	4 (44)
**Location**		
Rural	6 (60)	6 (67)
Urban	4 (40)	3 (33)
**Health Institution**		
Health Center	7 (70)	NA
Hospital/2nd level health center	3 (30)	NA
**Physician Specialty**		
General Practitioner	6 (60)	NA
Nephrologist/Internal Medicine	4 (40)	NA
**Years of experience**	19 (9–30)	10 (1–25)
**Interview length (minutes)**	41 (11)	18 (4)

Results are number (%) for all variables, except median (range) for years of experience and mean (sd) for interview length. NA: Not Applicable.

### CKD: prevalence, diagnosis and prognosis of a serious and increasing problem in Nicaragua

All interviewees stated that CKD is a serious problem. With the exception of one pharmacist, all interviewees were of the opinion that the number of CKD cases has increased each year, although four physicians suggested that this may be partially explained by improvements in monitoring and diagnoses.

All physicians indicated that the observation of decreased kidney function over time would be the basis for the CKD diagnosis. Seven of the ten physicians observed that patients with CKD had no or very little proteinuria. Six physicians relied on serum creatinine to classify a patient with CKD while four also calculated estimated glomerular filtration rate. Seven physicians indicated that, while ideal, kidney ultrasounds require referral to a hospital or private clinic and are usually not feasible. In terms of treatment, seven of the ten physicians said they do not have access to national or regional guidelines for the diagnosis and treatment of CKD and therefore relied on guidelines for other chronic diseases (hypertension or diabetes), their own familiarity with CKD, or, in one instance, an “ad hoc” protocol developed by local physicians. Three physicians who said they had access to CKD-specific diagnosis and treatment guidelines referred to protocol of Nicaragua’s Nephrology Association or the Ministry of Health, or international standards published by the National (USA) Kidney Foundation’s Kidney Disease Outcomes Quality Initiative. All physicians indicated that patient survival depends on stage of CKD at diagnosis, self-care, and nutrition as well as access to dialysis or transplant, neither of which are widely available in Nicaragua.

### CKD causes: strong sun, hard work, and… water?

All ten physicians and six of nine pharmacists described men as the most affected population. More than half of the physicians volunteered that the causes of the CKD epidemic did not include the traditional risk factors of diabetes or hypertension. Four physicians suggested that, while women experience CKD, the majority of cases in women can be explained by underlying risk factors. The affected population was most often described as under age 45.

Seven of the ten physicians identified agricultural workers (fruit pickers and workers on banana, cotton, melon, peanut, rice, and sugar plantations) as most affected by CKD. Eight of nine pharmacists explicitly identified what they thought was an occupational association, with one stating: “Those who are not diabetic are almost all males who work in the fields.” Other non-agricultural occupations mentioned included miners, construction workers and bricklayers.

Nine physicians referred to exposures at work, with seven identifying exposure to sun and heat as the major occupational factor associated with CKD. All ten physicians described many possible contributors to CKD, with six using the term “multi-factorial.” Chemicals used in agriculture were identified as a possible cause by half the physicians, and three talked about contaminated drinking water, with two referring specifically to heavy metals.

Water was the common denominator in eight of nine pharmacist responses regarding the causes of CKD; however, there were two distinct perspectives on the role of water. Four pharmacists thought that insufficient water intake was the primary problem, while four other pharmacists believed that drinking contaminated water was the problem. Three of the four pharmacists who thought dehydration was the problem also thought it was not the only cause, or else, as one said, “everyone would be dead.” Another pharmacist summarized beliefs about causes:

“I think that [CKD] is from the dehydration they get in the fields, their work is hard, hard, and they ruin themselves taking diuretics and further draining their body water.”

Other causes of CKD mentioned by more than one interviewee (in order of frequency) were the use of nephrotoxic medications, alcohol, and poor nutrition. Other possible contributors mentioned included leptospira infection and volcanic ash.

### “Chistata” and UTI: physician perspectives on their diagnosis and treatment, and associations with CKD

All physicians said that “chistata” is a colloquial term used to characterize a constellation of symptoms including “pain,” “burning” and the “urgent” need to urinate, with chistata approximating the clinical term dysuria. Nine physicians opined that dehydration is a probable cause of chistata, while eight also cited UTIs. There was disagreement among physicians as to how closely infection was related to chistata, with some equating the two, and at least one insisting, “they cannot just be considered synonymous.”

When queried further about UTI, four of the ten physicians claimed to diagnose UTI based on the results of a urine culture. However, two of these physicians acknowledged that they do not have the facilities to analyze a urine culture and, as one stated, it would be “at the cost of the patient.” Instead, these two along with five others admit to diagnosing UTI based on a urine exam indicating the presence of leukocytes, bacteriuria and/or nitrites via dipstick when available, under a microscope, or a combination of both practices. One physician suggested UTIs are probably over diagnosed:

“What happens is that we have been doing a bad management of the urinary tract infections… sometimes we consider that the appearance of white blood cells, leukocytes, in urine is enough. But it is not like that.”

When asked about the relationship among chistata, UTI and CKD, seven physicians said that UTIs may be associated with CKD. Five thought the connection had to do with repeated, non-treated or inadequately treated UTIs and regular use of broad spectrum antibiotics.

There was no agreement regarding the relationship of nephrolithiasis with CKD. Four physicians said that stones are not frequent, while six said they are very frequent. Three physicians named kidney calculi as a possible cause of chistata, with two associating them with “too concentrated urine” or “sandy urine” due to dehydration.

When asked how chistata is treated, seven physicians said they recommend oral rehydration solutions, and seven recommended urinary analgesics such as phenazopyridine or the antibiotic nitrofurantoin. Five physicians specified that they would not treat chistata without further tests. Antibiotics that they might then prescribe included fluoroquinolones, amoxicillin, and penicillin, with one physician naming aminoglycosides.

Treatment of UTIs included the same urinary analgesics and antibiotics enumerated for chistata plus other beta-lactam antibiotics and cephalosporins. One physician stated that gentamicin should be used only in very select cases, cautioning of its nephrotoxicity. When answering a question regarding the uses of gentamicin later in the interview, most physicians responded that it was reserved for severe UTI or sepsis patients. Two noted its nephrotoxicity, with one noting he sometimes had to use gentamicin due to the unavailability of other medications.

Eight physicians acknowledged the possible nephrotoxic effects of long-term intake of NSAIDs, with six saying they would not prescribe them (or would restrict their use) in CKD patients. However, two physicians said NSAIDS are prescribed for back pain.

### Pharmacist perceptions of UTI, Chistata, CKD and their treatment

Pharmacists did not make the distinctions that physicians did among the symptoms or conditions of low back pain, chistata, UTI, and CKD. Three pharmacists referred to CKD as an infection. Three said that UTI and chistata are the first step, or “warning,” of CKD. Three other pharmacists suggested that CKD results from UTIs that were either not treated or treated ineffectively.

When asked specifically about prescriptions filled for CKD, eight of nine pharmacists named phenazopyridine (not known to be nephrotoxic) in addition to antibiotics. One pharmacist offered the following: “For kidney insufficiency, doctors from health centers here commonly prescribe ampoules called gentamicin, trimethoprim-sulfa…” This contradicts what the majority of physicians indicated as per gentamicin prescription. For the most part, however, pharmacists described giving the same medications as the physicians for treatment of chistata and UTIs plus some antispasmodic drugs and NSAIDs. Pharmacists did not mention oral rehydration solutions.

Pharmacists’ responses revealed a range of opinions and practices regarding their role in the diagnosis and treatment process. On one end of the spectrum are those who would not prescribe anything until a patient is diagnosed by a physician, and on the other end are those willing to provide treatment directly. However, most said they would first recommend the patient obtain a urinalysis and have it interpreted by a physician. In some cases pharmacists offered to interpret lab results. Four pharmacists described the practice of selling antibiotics without a doctor’s prescription or a formal diagnosis. One pharmacist described the pressure to sell antibiotics over the counter with no diagnosis: “They*’*ll say, *‘*Why can*’*t you give me one directly? Why do I have to go and spend money to get an exam?*’* So I recommend them cefadroxil.”

Three pharmacists said that they would first recommend diuretics: “Furosemide is the most common, I sell it every day.” Recognizing that diuretics could further tax the kidney, another pharmacist explained:

“When I sell it, I tell people to drink plenty of fluids. But at the end, that is the decision of people, right? But I let them know that [the medication] can damage the kidney.”

Several physicians opined that pharmacists are enabling patients to self-medicate and selling potentially harmful medication: “The problem is that NSAIDs and gentamicin are sold in the markets of cities like candies” and “agricultural workers with back pain get shots of gentamicin by vendors in stores.” Taking diuretics for chistata was also described by two physicians:

“… these patients that normally have chistata are dehydrated most of the time, so what they do is take a furosemide to get rid of [chistata]. And later, they arrive with cramps, more dehydrated, and it becomes chaos.”

## Discussion

Interviews of physicians and pharmacists in Western Nicaragua were consistent with the existence of an epidemic of CKD, describing characteristics similar to those noted in the prevalence studies conducted in the region [10-13,22]. These include CKD being more frequent among men, starting in young adulthood, and associated with mainly agricultural work but also occurring in miners and construction workers. The sentiment that diabetes and hypertension cannot explain the CKD epidemic in Nicaragua was also expressed in the interviews. If this sentiment is based on empirical observations of physicians and pharmacists, these findings provide additional verification of the findings of local studies. The primary contribution of this study, however, is highlighting medication usage patterns described by physicians and pharmacists that have not previously been well characterized in cohort studies and government data, and which may lead to increased risk of CKD among younger men in this region.

Interviews indicate that patients with CKD, or at high risk for CKD, may be receiving nephrotoxic drugs for the syndromes of chistata and UTI. In particular, aminoglycoside antibiotics and chronic use of NSAIDs are associated with acute kidney injury (AKI) in a dose- and duration-dependent manner [23-25]. This association is particularly notable in the setting of volume depletion, which may be further aggravated due to the use of diuretics. Reports that attempt to quantify regional NSAIDs use vary widely, with self-reported use ranging from 10 to 75% of study populations [11,12,14].

Despite the media attention given to the potential role of agrichemicals in causing CKD, physicians and pharmacists were much more likely to cite exposure to heat, physical work and dehydration as key factors responsible for CKD; a common combination of exposures among men in this region and in Central America [17,22,26,27]. This opinion is consistent with local and regional studies, which find that CKD appears more common among occupations where strenuous work undertaken at high ambient temperatures is typical [9-13,28,29]. Although not a recognized cause of CKD, heat stress is associated with volume depletion and may also be associated with muscle damage (rhabdomyolysis), both of which may predispose individuals to AKI, particularly in the presence of nephrotoxic medications [26,27,30,31]. Critically, there is a growing body of literature that suggests that AKI, even if mild or if the serum creatinine recovers to baseline, may result in residual structural damage and ultimately progress to clinically recognized CKD [32-36] (See Figure 1).

Our interviews provide inconsistent yet important information regarding the perceived role of drinking water in CKD, with half the pharmacists believing that not drinking enough water leads to CKD, and the other half believing that CKD may be caused by drinking contaminated water. These beliefs, taken at surface value, would result in contradictory recommendations to area residents and workers. However, it is too early to say which view is correct and the truth may lie somewhere in between. Even if drinking water contaminants are not known to directly cause CKD, it cannot be concluded that drinking water quality is acceptable in various areas. Additionally, the emphasis of interviewees was that dehydration is due in part to perceptions of water and water consumption practices. The question remains, is hydration in general recommended or water consumption specifically? If lacking clean water, should workers and the general public be advised to hydrate with soda or fruit juices? What are the risks and benefits of hydrating with these high-sugar fluids compared with unclean water? To the extent that the contradictory views of pharmacists may represent those in the broader community, further examination of these questions would be beneficial before public health professionals design interventions with messages simply exhorting people to drink more water.

According to both physicians and pharmacists, UTI is one of the main causes of chistata. However, in medical practice elsewhere UTI is very uncommon among younger men with the rare cases typically associated with congenital urinary tract abnormalities or obstruction such as from nephrolithiasis. Urine cultures analyzed by our group from 47 male workers whose urine dipstick results were positive for leukocyte esterase or who had complained of dysuria within the past 24 hours were all negative for bacterial growth [37], supporting the impression of some physicians that UTI is likely over-diagnosed.

Exposures to agrichemicals, heavy metals, alcohol consumption and infectious agents were also mentioned in physician and pharmacist interviews and are the focus of additional causal hypotheses in the region [11-14]. In particular, infection by leptospirosis, although mentioned by only one interviewee, is considered an occupational hazard for people who work outdoors, including sugar-cane field workers, and a high seroprevalence of leptospirosis has been described in northwestern Nicaragua [38,39]. Manifestations of leptospirosis range from subclinical infection to severe disease accompanied by acute kidney failure. To date, there has been little research regarding the potential for leptospirosis infection to cause chronic tubular dysfunction or CKD in the absence of severe acute illness [40-42].

Limitations of our study include the inability to determine the truthfulness and accuracy of interviewee responses, and the small number of physicians and pharmacists interviewed. Qualitative studies are rarely designed to have statistically representative samples of the population, but for several of our questions we could have learned more about the general practice among physicians in particular if we had been able to conduct more interviews and achieved a higher degree of redundancy in the responses. However, these interviews have highlighted several potential contributors to CKD in Nicaragua including heat stress and use of potential nephrotoxic medications, supporting the plausibility of a multi-factorial cause of CKD. These findings also provide impetus for future cohort studies designed to examine such exposures and diagnoses.

### Recommendations

Many features of CKD in Central America require further elucidation. These include identifying the true cause of “chistata” and UTI-like symptoms, evaluating potential associations among heat stress, UTI diagnosis and nephrotoxic medication use, evaluating the role of systemic infectious diseases, and assessing the potential contribution of all of these factors to the development of CKD. However, until such time, the possible association between the occupational exposure to heat and the use of potentially nephrotoxic medications may be a beneficial focus of regional preventive health programs [43], including at the workplace, at the community level, and in health care settings. Surveillance systems of worker symptoms of heat exposure and workload, together with hydration practices, could be consolidated by governmental agencies, periodically assessed for lessons learned/best practices, and shared with employers. Similarly, there have been several assessments of water quality in specific areas. These assessments could be compiled with a similar analysis, identifying and addressing data gaps with further testing, so (a) public health professionals can make scientifically-based recommendations regarding consumption of water, and (b) local health agencies could come up with rapid and inexpensive processes to check for local variability (e.g., arsenic may be an issue in some areas, not in others). Overall, research into current hydration practices and promoters, and obstacles to greater hydration, could help shape public health recommendations.

Common self-medication reported by pharmacists and physicians of widespread (across the community and particularly workers) dysuria related symptoms with diuretics, NSAIDs and antibiotics could be the target of greater oversight and health protection measures from government agencies. The contradiction between pharmacists reporting that physicians prescribe gentamicin, and physicians reporting otherwise, could also be explored via observations at pharmacies where prescriptions are filled. Finally, we recommend increased and targeted education for pharmacists and physicians on the diagnosis and treatment of heat exposure symptoms, UTIs and CKD, along with strategies that may assist pharmacists when confronted with patients who demand medications that may be nephrotoxic.

## Conclusions

High mortality has been documented due to CKD among young adult men in Northwest Nicaragua, and specific areas of Central America including regions of El Salvador and Costa Rica, the cause of which remains unknown. This study identifies perceived causes of the CKD epidemic in Nicaragua by physicians and pharmacists working on the front line. Our analysis articulates perceptions of physicians and pharmacists that heat stress and subsequent volume depletion experienced by manual laborers plays a role in the frequent occurrence of dysuria-like symptoms, which are often treated with non-steroidal anti-inflammatory drugs, diuretics and antibiotics that may be further nephrotoxic.

## Competing interests

The authors declare that they have no competing interests.

## Authors’ contributions

Each author has participated in the work here presented and takes public responsibility for the content. Each author also contributed to the development of the interview guide, the interpretation of the findings reported in this manuscript, and drafts of the manuscript itself. Specifically, DRB is the Principal Investigator of the parent study of CKD in Nicaragua. MKS led the qualitative interview study design and analysis reported in this manuscript. ORR, a physician and specialist in preventive medicine and public health with experience in qualitative research, conducted the in-person interviews in Nicaragua and is responsible, along with MKS, for primary analysis of the results and initial drafting the article. JJA, who is located in Nicaragua, helped obtain local ethics approval, oversaw the recruitment strategy and fieldwork, and confirmed accurate translation of the interview guides into Spanish. JSK and DEW, both nephrologists, assisted in the interpretation of our findings and in drafting of the manuscript. All authors read and approved the final manuscript.

## Authors’ information

ORR, MD, MPH, is a preventive medicine and public health specialist pursuing her PhD at Universidad Autonoma de Madrid, Spain, and an international research scholar at Boston University School of Public Health, Boston, MA USA. DRB, DSc, is an Associate Professor of Epidemiology at Boston University School of Public Health, Boston, Massachusetts, USA. JJA, MD, MPH, is a public health expert and epidemiologist from Nicaragua, who leads the Boston University field research team. JSK, MD, is formerly a Professor of Medicine and Staff Nephrologist at the VA Boston Healthcare System and Boston University School of Medicine, and currently with the VA New York Harbor Healthcare System. DEW, MD MS, is Assistant Professor of Medicine at Tufts University School of Medicine and a Nephrologist at Tufts Medical Center, Boston, Massachusetts, USA. MKS, DSc, is an Assistant Professor of Environmental Health at Boston University School of Public Health, Boston, Massachusetts, USA.

## Pre-publication history

The pre-publication history for this paper can be accessed here:

http://www.biomedcentral.com/1471-2458/13/350/prepub

## Supplementary Material

Additional file 1 **Illustrative interview quotes.** Quotes by physicians and pharmacists that further illustrate results presented in manuscript. Click here for file
