# Supplementary material for: Chronic kidney disease in Nicaragua: a qualitative analysis of semi-structured interviews with physicians and pharmacists
Source: BMC Public Health. 2013 Apr 16;13:350. doi: 10.1186/1471-2458-13-350 (PMC3637184; doi:10.1186/1471-2458-13-350)
Supplement: Additional file 1 — Illustrative interview quotes. Quotes by physicians and pharmacists that further illustrate results presented in manuscript. [file 1471-2458-13-350-S1.pdf]

Additional file 1 – Illustrative interview quotes

CKD: A serious and increasing problem in Nicaragua
"In the last five years they've been dropping like flies, almost one person dead or very, very sick with this illness each day." (Pharmacist)<br><br>"More men are affected than women… This doesn't mean that women are not... but these are women who have a chronic pathology to begin with." (Physician)<br><br>"What caught my attention was that there are young people, very young, and that they are rapidly getting worse. We have them age 20, 23, 24, and I am telling you we have detected it in people that maybe have two years, and they have deteriorated, significant health deterioration…" (Physician)<br><br>"If we had family support… they are very cooperative, but they're people of few resources, they become like a burden for their family, and many of them are also patients who are alone, they're not retired, they're in poverty, so really, that kind of speeds up the suffering." (Physician)<br><br>"Well, I see dehydration, with faces like [scrunched up], uncomfortable. When they have had the illness for a while and are deteriorating, they look yellow, thin. There are others that swell. When cases like this come in, I send them to the health center to get a doctor." (Pharmacist)
**Causes of CKD(in Nicaragua)**
"Normally, they don't present symptoms [of CKD], but the symptoms that they do present with when they arrive for the first time, are generally heat strokes, people are working and have cramps, nausea, vomiting, with fever. …After re-hydrating them, people improve and the creatinine levels after three or four months can lower from 2.8 or 3, to normal ranges. And they get reintegrated to work, but they still suffer from heat strokes. So there are possible consequences from dehydration." (Physician)<br><br>"Patients have worked in agriculture - more than anything they have been exposed to the sun a lot, they have been exposed to dehydration, they work with sugar cane, they work in the cotton fields." (Physician)<br><br>"The majority are young men who have worked under the sun, under the heat, and maybe they haven't been drinking enough liquid when they have sunstroke or dehydration." (Physician)<br><br>"Men have other factors associated with deterioration of kidney function… the use and

abuse of substances, alcohol or drugs, strenuous exercise or work, not necessarily exposure to pesticides, but work in the fields, or work in areas with many hours exposed to the sun…" (Physician)<br><br>"Those who are not diabetic are almost all males who work in the fields…. The most affected ones are, well, from the rural counties who work with pesticides and insecticides…. And then there are those from the city who are diabetic." (Pharmacist)<br><br>"I insist that I believe there is an interstitial nephritis, caused by something in the environment that we have been unable to determine." (Physician)<br><br>"Well, now we have leptospira, which means renal damage and it is also a public health problem. We had patients admitted here with abnormal creatinine, with confirmed diagnosis of leptospira. That is frequent." (Physician)
**Perceptions of UTI, Chistata, CKD and their treatment**
"[With the general urine exam] we can have data that give us signals of a chronic kidney infection, just starting or advanced. CKD signs will be changes in the casts, a change in urine density; we can find a change in the pH, within the important phases, and the leukocyturias, that don't have an established etiologic cause. That should get our attention and start us to investigate." (Physician)<br><br>"Here people say, 'sell me something for chistata', the majority want you to sell them furosemide pills. These pills dehydrate them. It's my duty to explain to them that this pill is not for that. 'Take the fenazopridine (phenazopyridine) for that.' 'No because that stains me (the urine), give me the half white ones please… look, give me the white ones, if not I'll go shop somewhere else'." (Pharmacist)
